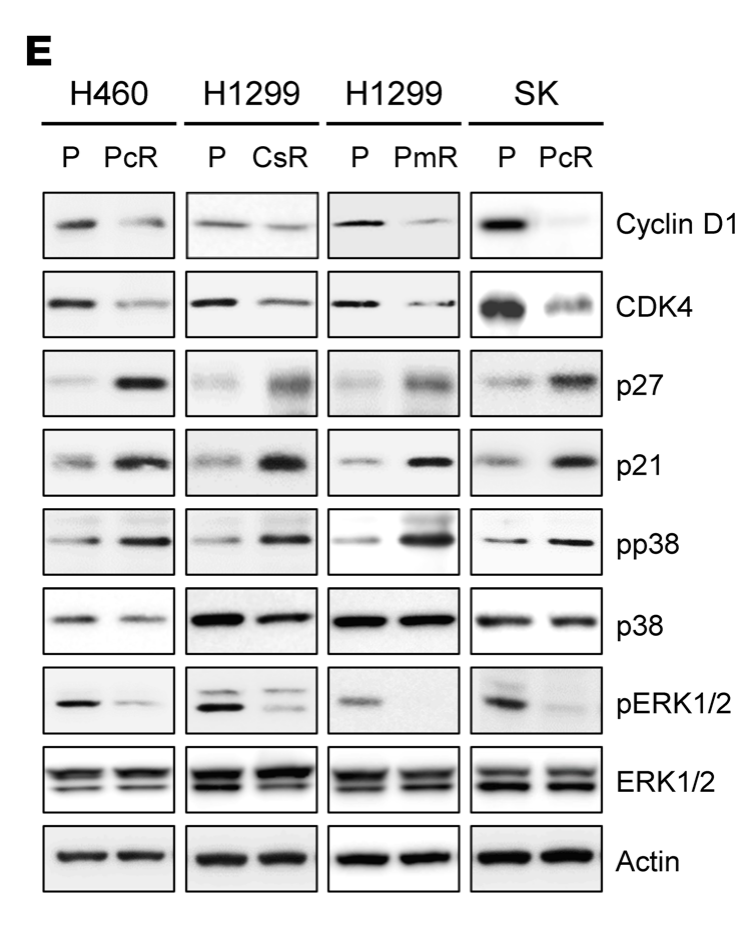# RGS2-mediated translational control mediates cancer cell dormancy and tumor relapse

**DOI:** 10.1172/JCI171901

**Published:** 2023-05-15

**Authors:** Jaebeom Cho, Hye-Young Min, Ho Jin Lee, Seung Yeob Hyun, Jeong Yeon Sim, Myungkyung Noh, Su Jung Hwang, Shin-Hyung Park, Hye-Jin Boo, Hyo-Jong Lee, Sungyoul Hong, Rang-Woon Park, Young Kee Shin, Mien-Chie Hung, Ho-Young Lee

Original citation: *J Clin Invest*. 2021;131(1):e136779. https://doi.org/10.1172/JCI136779

Citation for this corrigendum: *J Clin Invest*. 2023;133(10):e171901. https://doi.org/10.1172/JCI171901

The authors recently became aware of an inadvertent error in Figure 3E. In the original version of Figure 3E, the H1299 Cyclin D1 blot was incorrect and was the same as the SK Cyclin D1 blot. The correct figure is shown below. The HTML and PDF versions have been updated online.

The authors regret the error.

## Figures and Tables

**Figure:**